# *Helicobacter pylori* gene silencing *in vivo* demonstrates urease is essential for chronic infection

**DOI:** 10.1371/journal.ppat.1006464

**Published:** 2017-06-23

**Authors:** Aleksandra W. Debowski, Senta M. Walton, Eng-Guan Chua, Alfred Chin-Yen Tay, Tingting Liao, Binit Lamichhane, Robyn Himbeck, Keith A. Stubbs, Barry J. Marshall, Alma Fulurija, Mohammed Benghezal

**Affiliations:** 1 *Helicobacter pylori* Research Laboratory, Marshall Centre for Infectious Disease Research and Training, School of Biomedical Sciences, University of Western Australia, Nedlands, Western Australia, Australia; 2 Ondek Pty. Ltd., Marshall Centre for Infectious Disease Research and Training, School of Biomedical Sciences, University of Western Australia, Nedlands, Western Australia, Australia; 3 School of Molecular Sciences, University of Western Australia, Crawley, Western Australia, Australia; Children's Hospital Boston, UNITED STATES

## Abstract

*Helicobacter pylori* infection causes chronic active gastritis that after many years of infection can develop into peptic ulceration or gastric adenocarcinoma. The bacterium is highly adapted to surviving in the gastric environment and a key adaptation is the virulence factor urease. Although widely postulated, the requirement of urease expression for persistent infection has not been elucidated experimentally as conventional urease knockout mutants are incapable of colonization. To overcome this constraint, conditional *H*. *pylori* urease mutants were constructed by adapting the tetracycline inducible expression system that enabled changing the urease phenotype of the bacteria during established infection. Through tight regulation we demonstrate that urease expression is not only required for establishing initial colonization but also for maintaining chronic infection. Furthermore, successful isolation of *tet-*escape mutants from a late infection time point revealed the strong selective pressure on this gastric pathogen to continuously express urease in order to maintain chronic infection. In addition to mutations in the conditional gene expression system, escape mutants were found to harbor changes in other genes including the alternative RNA polymerase sigma factor, *fliA*, highlighting the genetic plasticity of *H*. *pylori* to adapt to a changing niche. The *tet*-system described here opens up opportunities to studying genes involved in the chronic stage of *H*. *pylori* infection to gain insight into bacterial mechanisms promoting immune escape and life-long infection. Furthermore, this genetic tool also allows for a new avenue of inquiry into understanding the importance of various virulence determinants in a changing biological environment when the bacterium is put under duress.

## Author summary

*Helicobacter pylori* is a bacterial pathogen that chronically infects half the global population and is a major contributor to the development of peptic ulcers and stomach cancer. *H*. *pylori* has evolved to survive in the stomach and one important adaptation is the enzyme urease. The bacteria cannot establish an infection in the host without this enzyme, and although widely postulated, the requirement of urease for chronic infection of the host has not been tested experimentally as conventional urease mutants are incapable of colonization. To overcome this constraint, a genetic system was introduced that allowed for the making of *H*. *pylori* strains in which urease expression could be turned off after the bacteria have colonised the stomach. We show for the first time that this enzyme is not only important for initial colonization but that it is also very important for maintaining chronic infection. We also show that if urease is turned off, the bacterium can mutate several different genes in order to restore urease expression. The genetic approach described here opens up opportunities to studying genes involved in the chronic stage of *H*. *pylori* infection to gain insight into how the bacterium is able to avoid clearance by the immune system and how it is able to adapt to changing biological environments.

## Introduction

The human gut pathogen *Helicobacter pylori* has coevolved with humans over thousands of years to dominate the gastric niche [1–3]. The majority of infected individuals (80–90%) carry and transmit *H*. *pylori* without any symptoms of disease [4, 5]. However, *H*. *pylori* infection causes chronic active gastritis that may develop into peptic ulceration (10–20%) or gastric adenocarcinoma (0.5–2%) [6, 7] causing a significant burden on public health [8–10]. *H*. *pylori* infection is persistent and clinical disease usually develops after many years of chronic inflammation and epithelial damage. Furthermore, due to increasing rates of antibiotic treatment failure [11, 12] there is a pressing need for further research into the bacterium’s mechanisms for persistence and immune evasion strategies. These are of particular importance to understanding *H*. *pylori* pathogenesis and to identifying novel targets for the development of new treatment options.

*H*. *pylori* is highly adapted to colonizing and surviving in the harsh conditions of the gastric environment. One key adaptation is the virulence factor urease. This multimeric enzyme, consisting of 12 UreA and UreB heterodimers, catalyses the hydrolysis of urea to produce CO_2_ and NH_3_, which acts to buffer the acidity of the local environment around the cell [13, 14]. Urease is abundantly expressed by *H*. *pylori* at levels exceeding that of any other known microbe [15] and is estimated to constitute 10–15% of the bacterium’s total protein content [16]. In addition, urease is essential for establishing colonization as *H*. *pylori* urease mutants are unable to infect the host [17–21]. Several lines of evidence suggest that urease plays a significantly greater role in infection than simple acid neutralization. Elevating the gastric pH to 7.0 was shown to be insufficient in permitting colonization by a urease negative strain [18]. In several *in vitro* studies urease and its catalytic products contributed directly to virulence. Ammonia produced by urease activity caused damage to the gastric epithelium by disrupting tight cell junction integrity [22, 23] and CO_2_ protected against the bactericidal activity of the nitric oxide metabolite, peroxynitrite, produced by phagocytes to kill engulfed bacteria [24]. Furthermore, several studies suggest that urease may directly interact with host epithelial and immune cells. Urease has been shown to bind to major histocompatibility complex (MHC) class II molecules on gastric epithelial cells thereby inducing cell apoptosis [25] and the UreB subunit can stimulate monocytes to release proinflammatory cytokines by binding to cell surface CD74, a MHC class II associated invariant chain [26, 27]. In addition, an *in vivo* study demonstrated that changes to the surface of the urease complex resulted in the eventual clearance of *H*. *pylori* infection in mice [28]. Loss of colonization was attributed to the disruption in urease mediated interactions between *H*. *pylori* and host cells as urease activity was unaffected by the mutation, ruling out loss of acid resistance or nitrogen assimilation [29] as contributing factors [28]. Clinical isolates maintain high urease activity even after years of chronic infection when the bacterium has established itself in the relatively neutral environment of its gastric niche implicating that ongoing urease expression is required for persistence. However due to the lack of appropriate genetic systems this hypothesis could not be tested experimentally.

The necessity of urease activity in establishing colonization hinders the study of its function during persistence when using conventional knockout mutants. The availability of a conditional urease mutant would overcome this constraint by permitting changes to the urease phenotype during an established infection. To conclusively determine if urease is indeed required after colonization is established, we generated conditional *H*. *pylori* urease knockout mutants using a tetracycline repressor (*tet*) based system [30]. This system controls gene expression by way of a tetracycline repressor (TetR) that binds to specific operator sequences (*tetO*) in the target promoter and silences transcription of the downstream gene. Expression of the target gene can be turned on by the administration of a potent tetracycline inducer, such as anhydrotetracycline (ATc) or doxycycline (Dox) [31]. This system was recently adapted to *H*. *pylori* and gene expression was regulated *in vivo* during active infection [32]. In the current study, we adapted this system to generate conditional urease mutants. We demonstrate that in an established infection, loss of urease expression is detrimental to the bacterial survival in the host. Strong selective pressure on the bacteria for continuous urease expression is further demonstrated by the emergence of escape mutants that successfully repopulated the mouse stomach six weeks after genetic silencing of urease was initiated.

## Results

### Construction and characterization of *urePtetO* promoters to drive urease expression

**Fig 1:**
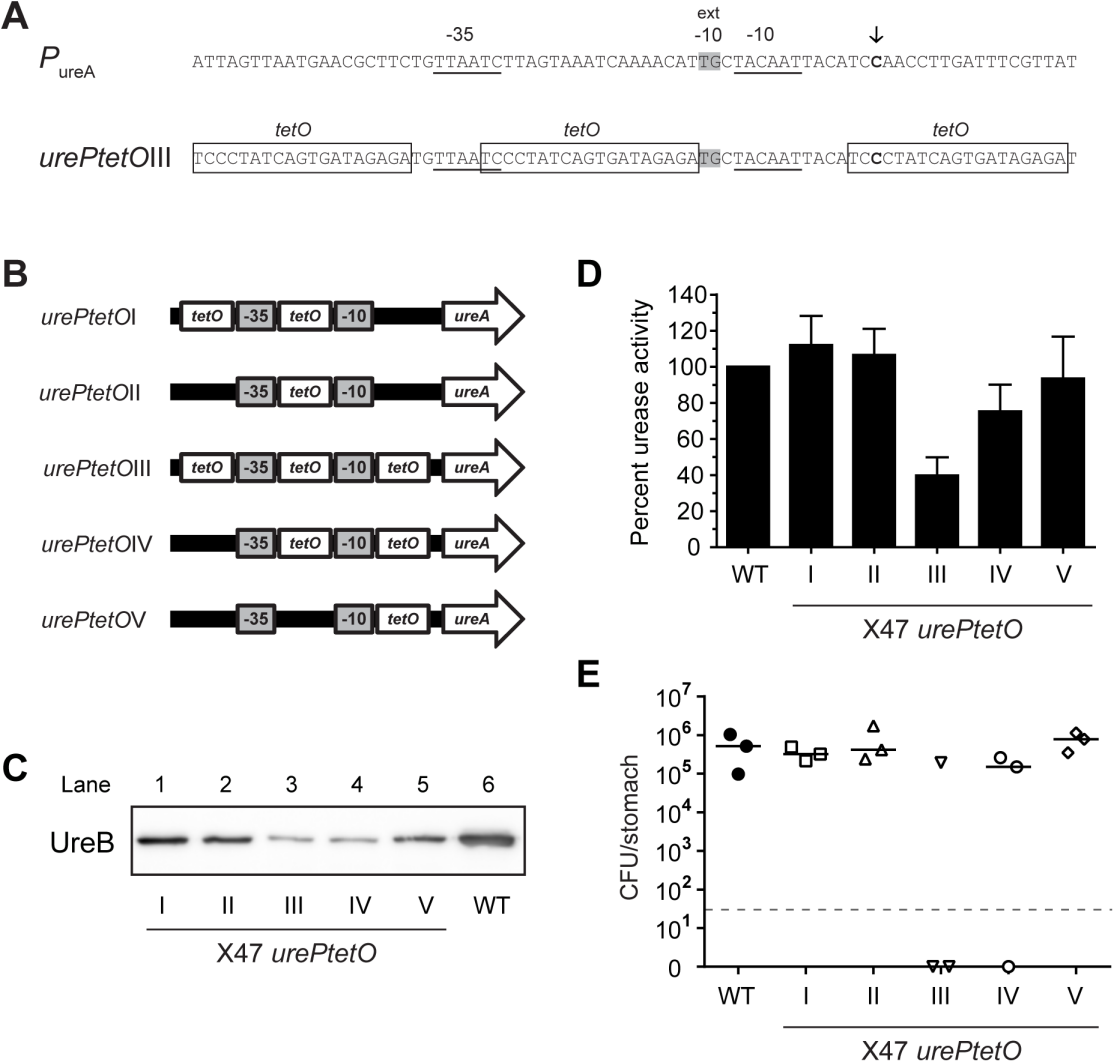
Structure and functionality of tetracycline responsive *ureA* promoters, *urePtetO*. (A) Nucleotide sequence (partial) of the wild-type *ureA* promoter, P_ureA_, and tetracycline responsive P_ureA_ derivative, *urePtetO*III. The -10 and -35 promoter sequences are underlined and the extended -10 region is shaded in grey. Boxes indicate *tet* operator (*tetO*) sequences. Arrow indicates the transcriptional start point (TSP). (B) Representative diagram of the *urePtetO* constructs. White *tetO* boxes indicate where the P_ureA_ promoter sequence has been replaced with *tetO* sequences. (C) UreB expression in X47 *urePtetO*(I-V) strains compared to wild-type X47. Fresh cultures of *H*. *pylori* grown on CBA plates were used to prepare *H*. *pylori* lysates. Equal amount of protein (~15 μg) was loaded into each lane and separated on a 10% SDS–PAGE gel. Lane 1, X47 *urePtetO*I (I), lane 2, X47 urePtetOII (II), lane 3, X47 urePtetOIII (III), lane 4, X47 urePtetOIV (IV), lane 5, X47 urePtetOV (V), lane 6, parent wild-type X47 (WT). (D) Urease activity in X47 *urePtetO*(I-V) strains (OND2018—OND2022) compared to wild-type X47. Fresh cultures of *H*. *pylori* grown on CBA plates for less than 24 h were collected and the urease activity for each strain was determined. Urease activity is expressed as a percentage of wild type X47 (WT) urease activity. The *urePtetO* construct is specified under each bar. All measurements were carried out in triplicate. Data are averages of three independent experiments and error bars represent standard deviations. (E) Two week colonization of C57BL/6J mice by X47 *urePtetO*(I-V) strains compared to wild-type X47 (WT). Modifications to the *ureA* promoter did not prevent colonization. Colonization studies were done without prior adaptation of X47 *urePtetO* strains to mice. Horizontal bars represent median bacterial load per group (n = 3) and points plotted represent colonization density for each individual animal. Detection limit was < 50 CFU per stomach (dotted horizontal line). Gastric specimens without *H*. *pylori* re-isolation are shown as null.

The urease structural genes, *ureA* and *ureB*, encoding for the 27 kDa UreA and 62 kDa UreB urease subunits, are transcribed as a single operon under the control of the *ureA* promoter, P_ureA_ [33]. To regulate the expression of urease in *H*. *pylori*, we placed the operon under *tet* control. Based on previous mutational studies of P_ureA_ [32, 34], the promoter was mutated to incorporate one or more *tetO* sequences to generate a series of different P_ureA_ derivatives, *urePtetO*(I-V) (Fig 1A and 1B). These *tet-*promoter constructs were made using PCR based techniques and used to replace the native chromosomal P_ureA_ by allelic replacement. This strategy involved first generating a recipient *H*. *pylori* strain in which P_ureA_ and *ureA* has been replaced with a *rpsL-cat* cassette. The urease negative recipient strain was then naturally transformed with the *urePtetO* PCR constructs to generate strains with *tetO* modified P_ureA_ derivatives and a restored *ureA* gene. *H*. *pylori* strains harbouring these constructs were characterized to identify a *tet*-promoter construct with regulatory properties that would permit the appropriate level of complementation to ensure colonization yet could be sufficiently silenced to prevent infection. The functionality of these *tet-*promoters was first assessed in the wild-type background for their ability to drive urease expression by measuring urease enzymatic activity, UreB expression and mouse colonization.

The *urePtetO* constructs were introduced into the wild-type X47 strain, replacing the chromosomal P_ureA_ and generating X47 *urePtetO* strains (OND2018—OND2022). The urease expression level and the urease enzymatic activity of these strains under standard growing conditions was measured and compared to the parent strain (Fig 1C and 1D). Despite expressing less urease, as determined by immunodetection of UreB in the total cell lysate (Fig 1C), the urease activity measured in strains transformed with *urePtetO*I, *urePtetO*II and *urePtetO*V was found to be comparable to wild-type (Fig 1D). In comparison, the urease activity in strains transformed with *urePtetO*III and *urePtetO*IV were reduced by 60% and 25% respectively, which was also accompanied by substantially reduced amount of UreB in the total cell lysate (Fig 1C).

Given that replacement of the wild type P_ureA_ with the *urePtetO* promoters resulted in reduced urease expression which concomitantly may reduce their ability to establish infection, C57BL/6J mice were challenged with strains X47 *urePtetO*(I-V) (OND2018—OND2022) to assess if urease expression in these strains was still sufficient to facilitate colonization. All five X47 *urePtetO* strains were successfully re-isolated from mouse stomachs two weeks after oral challenge (Fig 1E). The infection rate and bacterial load in mice challenged with strains X47 *urePtetO*I, X47 *urePtetO*II and X47 *urePtetO*V was comparable to the control group challenged with the wild-type strain. However the infection rates were decreased for groups challenged with strains of lower urease activity, with strain X47 *urePtetO*III displaying a major defect in colonization. To verify that colonization had not been established due to mutation or reversion of *urePtetO*, the *ureA* promoter region of re-isolated strains was sequenced which confirmed that the sequences of the *urePtetO* constructs remained unaltered after passage through mice.

### *Tet*-regulation of urease expression in *H*. *pylori*

After establishing that the *tetO* modifications to the *ureA* promoter did not abrogate colonization per se, we evaluated if these promoters could regulate urease expression in a tetracycline dependent manner. All five *ureA* promoter derivatives were transformed into a X47 recipient strain that expressed TetR under the control of the strong *flaA* promoter [32]. The resulting strains, X47 *mdaB*::*ptetR*4, *urePtetO*(I-V) (OND1954—OND1958), did not express urease when grown on standard CBA plates however urease expression could be induced when grown on CBA plates containing 50 ng/ml anhydrotetracycline (ATc) (S1 Fig). These results demonstrated that TetR effectively silenced the *tet*-promoters in these strains.

### Characterization of *urePtetO* regulated urease expression and activity *in vitro*

**Fig 2:**
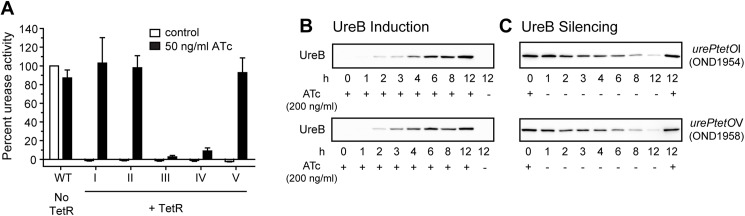
*Tet* regulation of urease activity and expression in *H*. *pylori*. (A) Urease activity assays of conditional urease mutants. Bacteria were cultured in the absence (white bars) or presence of 50 ng/ml ATc (black bars) for 48 h and fresh bacteria cultures were collected and used for activity assays. Five conditional *H*. *pylori* urease mutant strains, each harbouring a different *urePtetO* promoter, were tested: OND1954 (I), OND1955 (II), OND1956 (III), OND1957 (IV) and OND1958 (V). Urease activity is expressed as a percentage of wild-type X47 grown under standard conditions (WT). All measurements were carried out in triplicate. Data are averages of three independent experiments and error bars represent the standard deviation. (B+C) Time course of TetR-controlled expression of UreB in conditional urease mutants OND1954 and OND1958. (B) TetR-controlled induction of UreB expression. Bacteria were transformed as indicated and grown in HI media to mid-log phase (OD_600_ = 0.5) before addition of 200 ng/ml of ATc. Aliquots of induced cultures were taken at indicated time points. *H*. *pylori* lysates were separated on a 10% SDS–PAGE gel. Equal amount of protein was loaded into each lane. Far right lane, control culture with no ATc added. (C) Time course of TetR-controlled silencing of UreB expression. Bacteria were transformed as indicated and grown on CBA plates containing 200 ng/ml ATc for 48 h. Fresh bacteria were collected, washed with PBS and used to inoculate fresh HI media at a starting OD_600_ = 0.5 and grown for 12 h. Aliquots of each culture were taken at indicated time points. *H*. *pylori* lysates were separated on a 10% SDS–PAGE gel. Equal amount of protein was loaded into each lane. Far right lane, control culture with 200 ng/ml ATc, maintaining UreB induction.

Regulation of *urePtetO* promoters by TetR in conditional urease knockout strains X47 *mdaB*::*ptetR*4, *urePtetO*(I-V) was assessed using the urease enzymatic activity assay. Bacteria were cultured in the absence or presence of 50 ng/ml ATc for two successive passages and then collected for analysis. When strains were cultured in the absence of ATc, urease activity was below the detection limits of the assay (2 U/ml of Type III urease from Jack bean) (Fig 2A). For strains grown in the presence of ATc, urease activity in strains X47 *ptetR*4; *urePtetO-*I, -II and -V was induced to wild type levels, while the urease activity for strains X47 *ptetR*4; *urePtetO-*III and -IV remained below 10% of wild-type activity. These results demonstrated that by using the appropriate *tet-*promoter urease activity can indeed be regulated by the presence of a small molecule inducer, confirming the generation of conditional *H*. *pylori* urease knockout mutants.

The *tet-*responsive promoters *urePtetO*I and *urePtetO*V have different genetic architectures (Fig 1B) and upon induction also promoted the greatest urease expression levels amongst the tested strains. Based on these results the regulation of these two promoters was further characterized. The kinetics of *urePtetO* induction and repression was analysed in strain X47 *ptetR*4; *urePtetO*I (OND1954) and X47 *ptetR*4; *urePtetO*V (OND1958) by immunodetection of the UreB protein (Fig 2B and 2C). After addition of 200 ng/ml ATc to the culture medium, UreB protein expression increased over time and reached maximum levels after 12 h and 8 h for *urePtetO*I and *urePtetO*V, respectively (Fig 2B). Withdrawal of ATc from induced cultures led to a significant decrease in UreB protein levels within 3 h, demonstrating that both *urePtetO*I and *urePtetO*V were quickly silenced (Fig 2C) and that the UreB protein was turned over efficiently, falling to the threshold of detection within 12 h.

### Establishing a conditional urease knockout infection model

With the knowledge that tetracycline dependent regulation of urease expression was attainable *in vitro* we next turned our attention to establishing a mouse model of infection. Based on previous studies involving *in vivo tet*-systems [35–37] the inducer molecule doxycycline (Dox) was first used as a model inducer to identify a maximal dosage of material that could be tolerated by the bacterium *in vivo*. We found that wild-type X47 could still infect mice when the animals were supplemented with up to 10 mg/l of Dox in their drinking water. Colonization by wild-type X47 was severely attenuated at 100 mg/l of Dox and bacteria could not be reisolated at 1000 mg/l of Dox supplement (S2A Fig). Furthermore, strain X47 *ptetR*4; *urePtetO*I (OND1954), which emerged as the prime conditional urease mutant candidate from the *in vitro* studies, was tested to verify its ability to establish initial colonisation and then used to optimise the dosage of inducer molecule to regulate urease expression *in vivo*. OND1954 was only capable of establishing infection in C57BL/6J mice when the animals received Dox supplementation in their drinking water, demonstrating that urease is essential for OND1954 to establish colonization in the mouse infection model (S2B Fig). Addition of Dox supplement at 1 mg/l supported colonization of OND1954 and although attenuated, the conditional mutant was also isolated from animals supplemented with Dox at 5 mg/l and 10 mg/l.

**Fig 3:**
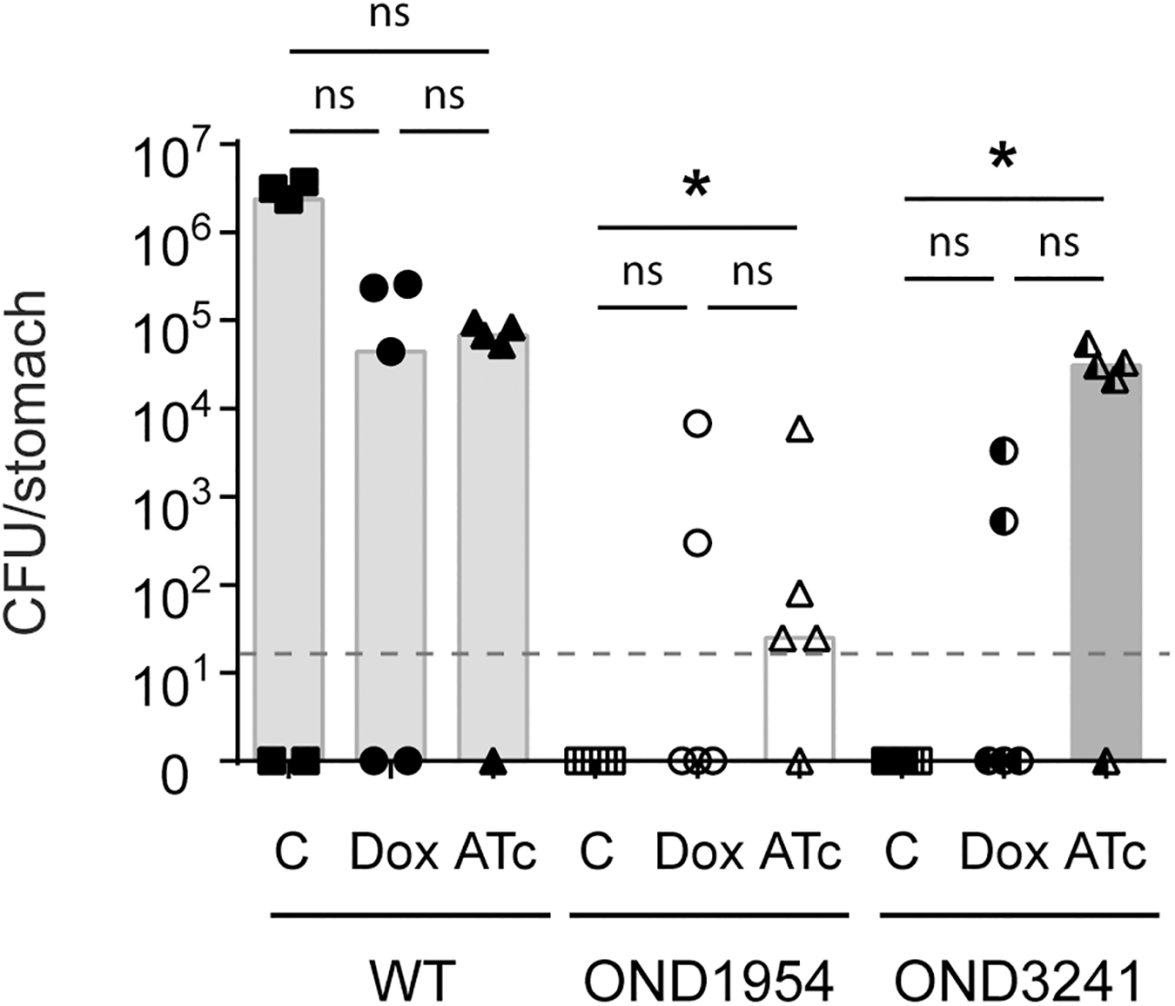
Infection of conditional urease mutant is dependent on tetracycline inducers. Optimization of *in vivo* model for *tet*-regulated urease expression. Mice groups were supplemented without (C) or with 5 mg/l of either Dox or ATc in their drinking water. Animals were orally challenged with wild-type strain X47, with pre-induced OND1954 or a pool of pre-induced clones of OND1954 re-isolated from mice (OND3241A-E). Animals were sacrificed two weeks after oral challenge. Bars represent median bacterial load per group (n = 5) and points plotted represent colonization density for each individual animal. Detection limit was < 50 CFU per stomach (dotted horizontal line). Gastric specimens without *H*. *pylori* re-isolation are shown as null. Statistical analysis was performed to test if Dox or ATc treatment influenced the infection rate of any of the given bacterial strains. Treatment did not significantly impact on the infection rate of the wild-type strain. However ATc supplementation significantly increased the infection rate for OND1954 and OND3241 (* p < 0.05). Unpaired two sided Fisher’s exact test followed by Bonferroni correction was used for pairwise testing of the infection status.

Having identified a minimal supplement dose of Dox inducer, we then sought to complete the infection model by investigating two more important factors; the use of the less toxic tetracycline derivative ATc, and attempting to improve the robustness of the conditional urease mutant strain. The original wild-type X47 strain underwent four consecutive transformations to generate the conditional urease mutant OND1954 and consequently the strain may have accumulated secondary mutations that would decrease its fitness *in vivo*. To address this, the output clones of OND1954 isolated from three individual mice were collected and each clone was verified to be a conditional urease mutant. These clones were then pooled, OND3241(A-E), and used in subsequent infection studies to test if passage though mice led to improved infection rates. Mice were challenged with either the wild-type strain, the original conditional mutant OND1954 or the mouse passaged urease conditional mutant OND3241, and supplemented without or with 5 mg/l Dox or ATc. Colonization of OND3241 remained dependent on inducer supplement and using ATc instead of Dox resulted in an improved infection rate and bacterial load in the infection model (Fig 3).

### Silencing urease during active infection

**Fig 4:**
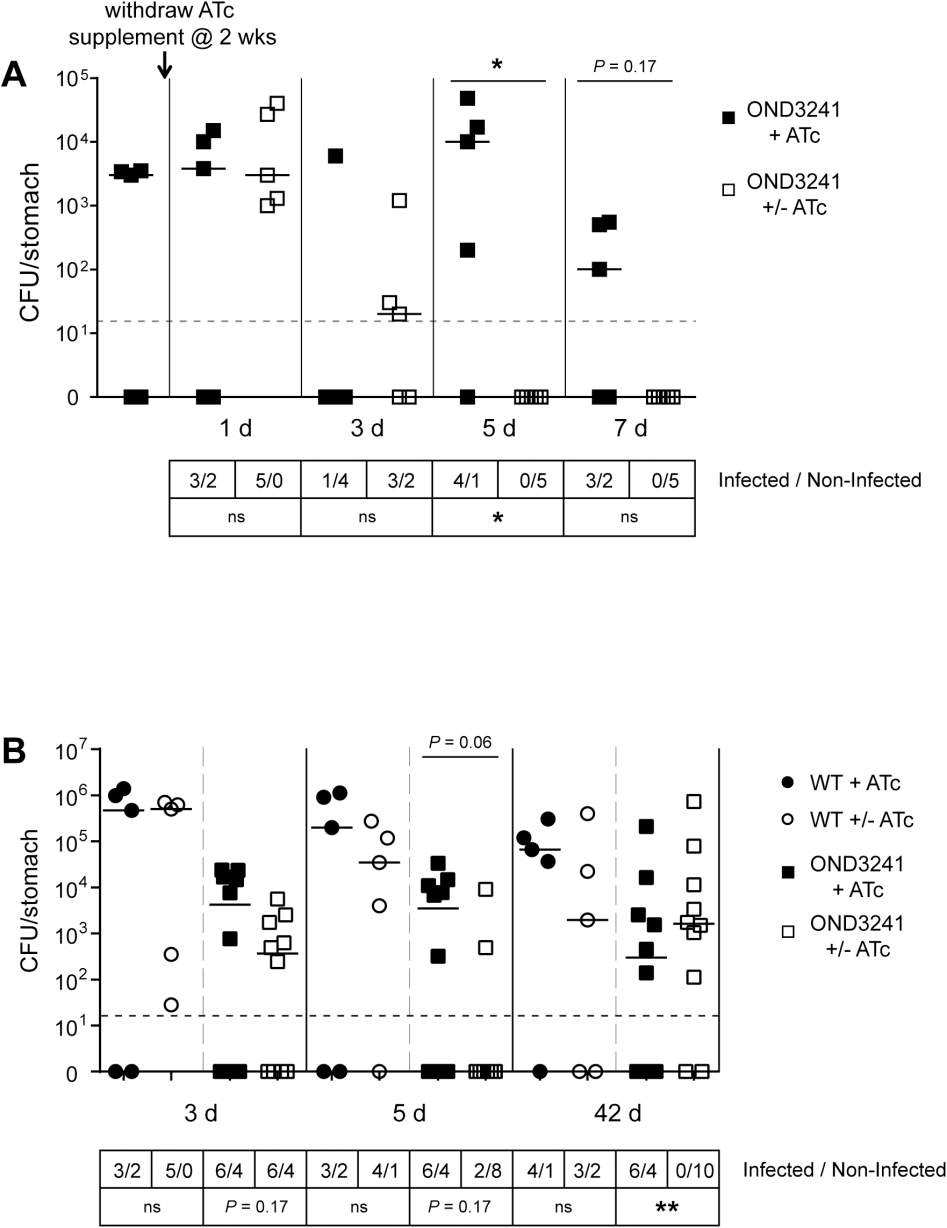
Tet-regulated silencing of urease expression during active infection. Urease expression in conditional urease mutant strains OND3241 was induced with 50 ng/ml ATc for 48 h prior to oral challenge. (A) Mice were orally challenged with pre-induced OND3241 and supplemented with 5 mg/l ATc for two weeks. ATc supplement was maintained (+, ■) or withdrawn (+/-, □) and animals were sacrificed 0, 1, 3, 5 and 7 days later. Bars represent median bacterial load per group (n = 5) and points plotted represent colonization density for each individual animal. Detection limit was < 50 CFU per stomach (dotted horizontal line). Gastric specimens without *H*. *pylori* re-isolation are shown as null. (B) Mice challenged with wild-type X47 (WT, ●) or pre-induced OND3241 were supplemented with 5 mg/l ATc for two weeks. After this two week period, ATc supplement was maintained (+, ■) or withdrawn (+/-, □) and animals were sacrificed 3, 5 and 42 days later. Bars represent median bacterial load per group (n = 10) and points plotted represent colonization density for each individual animal. Detection limit was < 50 CFU per stomach (dotted horizontal line). Gastric specimens without *H*. *pylori* re-isolation are shown as null. Statistical analysis using the unpaired two-sided Mann-Whitney test are indicated above the compared groups (* indicates that the geometric value of the (+/-, □) group is significantly different (p ≤ 0.05) from the (+, ■) group of the same time point) and unpaired two-sided Fisher’s exact test analysis of the infection rate by the conditional mutant is presented below the compared groups (* p< 0.05; *** p < 0.001). For the day 42 time point in panel B, as all the analysed colonies isolated from the (+/-, □) group were found to be escape mutants, none of the animals were considered infected with the conditional mutant resulting in a 0/10 infection rate.

Having established an infection model in which the *H*. *pylori* urease phenotype could be regulated *in vivo*, we proceeded to investigate what effect *tet*-mediated silencing of urease expression had on established *H*. *pylori* infections. Mice were challenged with the conditional strain OND3241 and provided with 5 mg/l ATc supplement to establish infection. After two weeks, the supplement was withdrawn and the animals were sacrificed at indicated time points. The conditional *H*. *pylori* urease mutant could still be isolated on days 1 and 3 after supplement withdrawal, however on days 5 and 7 the bacterial load had decreased to below our detection limit (Fig 4A). This data demonstrated for the first time that continuous urease expression is required by *H*. *pylori* to maintain colonization even after the bacteria have become established in the gastric niche.

### Isolation of escape mutants demonstrates strong selective pressure for urease expression

To test if *H*. *pylori* were under selective pressure to overcome *tet*-regulation the suppression experiment was repeated and the animals were sacrificed at a much later time point. Mice were challenged with either wild-type X47 or OND3241 and provided with 5 mg/l ATc supplement to establish infection. After two weeks, the supplement was withdrawn from half the groups (both OND3241 and wild-type) while the remaining groups were maintained on ATc supplement and the animals were sacrificed at different time points (Fig 4).

No differences in bacterial load or infection rate was observed for animals infected with wild-type X47 over the course of the experiment demonstrating that long-term ATc supplement (5 mg/l for 8 wks) does not interfere with *H*. *pylori* infection (Fig 4B). Animals challenged with OND3241 and maintained continuously on ATc supplement had a consistent infection rate of 60% (Fig 4B). *In vitro* tests confirmed that bacteria re-isolated from these groups remained conditional urease mutants, even after a total infection time of 8 weeks. Withdrawal of ATc supplement from the animal groups challenged with OND3241 resulted in reduced infection load on days 3 and 5 and, although not completely cleared to below our detection limit in all animals, the infection rate had decreased to 20% on day 5. However, when mice challenged with the OND3241 were left in the absence of ATc for 42 days, the bacterial load and the infection rate had increased resulting in 80% of the animals bearing bacteria in the stomach above our detection limit. Importantly, unlike the bacteria re-isolated at the earlier time points (day 3 and day 5), *H*. *pylori* re-isolated from this last group of mice were all urease positive and they were no longer conditional urease mutants as tested qualitatively *in vitro*. This result revealed that the strain was under selective pressure to restore urease expression.

In an effort to identify possible genetic mutations to overcome *tet*-regulation of the urease operon, whole genome sequencing of the original conditional urease mutant strain OND1954, the individual clones of input strain OND3241(A-E), and 34 output strains recovered at day 42 (5 conditional urease-negative strains and 29 urease-positive *tet*-escape mutants) was undertaken. Sequence data were mapped against reference strain OND1954 and variants specific to *tet*-escape mutants were identified (S1 Table). Sequence analysis of the *ureAB* locus, including the upstream regulatory region, revealed no changes between OND1954, OND3241 and the *tet-*escape mutants. Interestingly, the *tet-*escape mutants harboured non-synonymous substitutions or frameshift mutations in at least one of the following genes, *tetR* and the flagellar biosynthesis genes *fliA*, *fliE* and *flgE*. These data reveal that *tet*-regulation was overcome in the *tet-*escape mutants not by altering the *tetO* binding sites but through affecting the repressor protein.

## Discussion

A set of conditional urease mutants were generated to demonstrate for the first time that urease expression is essential in the persistence stage of *H*. *pylori* infection, which broadens our understanding of the role of this enzyme during chronic infection.

Genetic manipulation of P_ureA_ to place urease under *tet*-control led to a decrease in the basal levels of urease expression for all *urePtetO* constructs tested. However, under the conditions tested, strains transformed with *urePtetO*I, *urePtetO*II and *urePtetO*V were found to have comparable urease activity to that of wild-type. This data can be reconciled as it has been reported that under neutral *in vitro* growth conditions without added nickel, such as the growth conditions used in this study, a significant amount of urease in wild-type strains is in the inactive apoenzyme form and only a minor fraction of urease is active [38–40]. Urease activity in *H*. *pylori* is highly controlled and is modulated through several different mechanisms in response to various environmental cues [39]. Since our goal was to establish a working *in vivo* model, we decided to directly test if the decrease in urease expression could be tolerated by the bacteria by assessing if the *urePtetO* strains were capable of colonizing the murine stomach.

Interestingly, when analyzing the *in vivo* colonization data from the X47 *urePtetO* strains (OND2018—OND2022) a positive correlation between infection rate and *in vitro* urease expression and activity but not to bacterial load in colonized animals was observed (Fig 1E). This finding suggests that for initial colonization of the murine stomach the amount of urease activity is an important factor likely due to the fact that the bacteria need to withstand the acidity of the gastric lumen until they reach their gastric niche, deep into the gastric mucus near the epithelial surface. However, once the bacteria are established within their environmental niche, although urease is still required for growth, the level of urease expression may be less important for maintaining colonization as mice colonized with strains transformed with *urePtetO*III and *urePtetO*IV had a similar bacterial burden compared to mice infected with strains expressing more urease.

Infection of the mouse host by the conditional urease mutant was strictly dependant on supplementation with a tetracycline inducer, confirming that genetic regulation of urease expression was stringent enough to prevent colonization. Furthermore, in the induced state, *tet-*mediated expression of urease was sufficient to allow and maintain infection by the conditional urease mutant. Withdrawal of the supplement resulted in clearance of the bacterium within 5 days. This is in line with the slow shut-off observed in other mouse models using *tet*-based regulation systems which has been attributed to the persistence of doxycycline in tissues [41, 42].

Notably the longer time of clearance of the bacterium *in vivo* provided the opportunity for the emergence and selection of *tet*-escape mutants. *H*. *pylori* possess several mechanisms, such as an error prone PolA [43] and efficient DNA homologous recombination and transformation systems [44, 45], that permit the bacterium to undergo rapid microevolution to adapt to changing environments in its specific host [46, 47]. The emergence of *tet-*escape mutants in this study suggests that there is strong selective pressure on the bacterium for continuous urease expression to maintain chronic infection. Whole genome sequence analysis of the *tet-*escape mutants identified several different mutations that likely explain how *tet*-regulation of the *ureAB* operon was overcome.

One group of escape mutants had missense or nonsense mutation within the *tetR* gene. TetR is a finely tuned transcriptional regulator [31] and therefore most amino acid changes are likely to have deleterious effects to the function of TetR by inhibiting repressor dimerization or DNA binding [48–50]. Interestingly, another group of *tet-*escape mutants harbored either amino acid substitutions within the DNA binding domains of the alternative RNA polymerase sigma factor FliA (σ^28^) or a truncated FliA due to nonsense mutation and frameshift alteration. FliA controls the transcription of some late flagellar genes (class 3) including the flagellin subunit, *flaA* [51, 52]. Previous studies have reported that *H*. *pylori fliA* mutants have no detectable *flaA* transcript and have truncated flagella [51]. In the conditional *H*. *pylori* urease mutants generated in this study, the transcription of *tetR* is driven by a *flaA* promoter. Therefore, it is reasonable to suggest that the *fliA* mutations identified in the *tet-*escape mutants likely affect the transcription of *tetR* from P_fla_ and consequently release the *ureAB* operon from *tet*-regulation. Additionally, some *tet*-escape mutants acquired non-synonymous mutations in *fliE* and *flgE*, genes that encode for components of the flagellum hook-basal body complex. Flagellar biosynthesis is a highly ordered and regulated process and transcription of late flagellar genes by *fliA* proceeds only once the hook-basal body complex is complete [53, 54]. Mutation of *fliE* has been shown in other studies to affect the regulation of *fliA* dependent genes, leading to a two-fold reduction in *flaA* transcript levels [54]. Thus mutation of *fliE* and *flgE* may indirectly impact on the transcription of *tetR* from P_flaA_ through the negative control of FliA [51, 53]. Motility is essential for *H*. *pylori* colonization [19, 55, 56], and therefore the isolation of *tet*-escape mutants with mutations in genes involved in flagellar biosynthesis (*fliA*, *fliE* and *flgE*) raises interesting questions. During the chronic stage of infection, once the bacteria are established in their environmental niche and have adhered to gastric epithelial cells [57], can motility be compromised in preference for improved urease expression? Further investigations with the appropriate conditional mutants are necessary to better understand the escape and in particular to test whether motility is still required once colonization is established. Another question is whether there are other mutations acquired by the *tet*-escape mutants that could compensate for mutations in *fliA*, *fliE* and *flgE*? In-depth analysis of the whole genome sequencing data together with mutational studies needs to be undertaken to discern how these *tet*-escape mutants are able to survive in the host.

The sequencing results underline the importance of urease for *H*. *pylori* to maintain persistence infection and reveal the high selective pressure for continuous expression of urease even after colonisation is successfully established. The mutations identified in the *tet*-escape mutants add support to the hypothesis that the genomic plasticity of *H*. *pylori* is an important mechanism for adaptation to new and changing environments [58]. Furthermore these findings also highlight the potential of using *tet*-based genetic tools together with whole genome sequencing to study *H*. *pylori* genetic plasticity and adaptation in a changing biological environment when the bacterium is put under duress.

Using the conditional mutant, we have demonstrated that urease is essential for chronic infection and that repression of urease expression results in the loss of bacterial load within 5 to 7 days. The availability of these tools now allows for new questions to be asked regarding the reason behind the relatively rapid loss in colonization. One reason may be that the loss of urease activity results in the bacteria being more susceptible to clearance by phagocytic cells [24]. Another possibility is that the loss of urease activity negatively affects *H*. *pylori* ability to swim through gastric mucus, as the bacteria would no longer have the ability to decrease the viscosity of mucus through the elevation of local pH [59], and consequently are cleared due to turnover of the mucus lining. The conditional urease mutant will serve as a valuable tool in further studies that pursue this line of investigation.

In this study, *H*. *pylori* conditional urease mutants were generated by placing the expression of the urease subunits, UreA and UreB, under *tet-*control and have permitted the first direct testing of the hypothesis that urease is required by *H*. *pylori* for chronic infection. Furthermore, eventual escape from *tet*-regulated urease expression by *H*. *pylori* demonstrates that there is a very strong selective pressure on the bacterium to maintain urease expression during infection. Our data validates urease as a good target for therapeutic intervention. The conditional urease mutants generated here can also be used to gain more detailed insight into the role of urease in the persistence stage of infection including its interactions with MHC class II molecules [25], induction of proinflammatory cytokine [27] and its potential role in motility [59]. Furthermore, this study demonstrates the need for conditional mutants, generated by using genetic tools such as the *tet-*system, to study *H*. *pylori* virulence factors, persistence and the bacterium’s influence on the host microbiota.

## Materials and methods

### Bacterial strains and culture conditions

*H*. *pylori* X47 strains used in this study are listed in S2 Table. Bacteria were grown at 37°C under microaerobic conditions on Columbia blood agar (CBA) plates containing 5% horse blood and Dent’s antibiotic supplement (Oxoid). When appropriate, antibiotic selection was carried out by supplementing media with chloramphenicol or streptomycin at a final concentration of 10 μg/ml. Microaerobic conditions were established in sealed jars using the Anoxomat MarkII system (Mart Microbiology B.V., the Netherlands) after one atmosphere replacement with the following gas composition N_2_:H_2_:CO_2_, 85:5:10.

### Genetic manipulation and *H*. *pylori* strain construction

All genetic manipulation of *H*. *pylori* strains was done using genomic insertion and replacement of a counter-selectable *rpsL-cat* cassette [60]. The use of the counterselectable streptomycin susceptibility (*rpsL*-based) system requires a host strain that possesses a streptomycin-resistant phenotype [61]. The *H*. *pylori* X47 host strain is naturally streptomycin-resistant and no modifications to this strain were required. The genotype of all mutants was confirmed by PCR and/or DNA sequencing.

### Oligonucleotides

Oligonucleotides used in this study are listed in S3 Table.

### Construction of the *H*. *pylori* tetracycline responsive *urePtetO* promoters

To place *ureA* and *ureB* under *tet* control, wild-type nucleotide sequences flanking the -35 and -10 promoter regions of the *ureA* promoter, P_ureA_, were replaced with *tetO* sequences to generate five derivatives of P_ureA_, *urePtetO*(-I through -V) (Fig 1A and 1B). These promoter constructs were used to replace the native urease promoter, using the two-step *rpsL-cat* based transformation approach.

### Construction of *ureA*::*rpsL-cat* recipient strain

A construct composed of the counterselection cassette flanked by DNAs homologous to regions of the *ureA* locus, *ureA*::*rpsL-cat*, was made by SOE PCR [62, 63] (S3 Fig) and used to generate recipient strains in which P_ureA_ and *ureA* were replaced with *rpsL-cat*. Two 1 kb regions flanking P_ureA_ and *ureA* (HP0073), were amplified from 26695 genomic DNA using primers ureArcat1 and ureArcat2, and ureArcat3 and ureArcat4 respectively. The *rpsL-cat* selection cassette was amplified using primers ureArcat5 and ureArcat6. Nested primers ureArcat7 and ureArcat8, were used to generate and amplify a final 3.4 kb PCR product, *ureA*::*rpsL-cat*. Natural transformation of the *H*. *pylori* strains with the *ureA*::*rpsL-cat* PCR construct was performed to obtain the recipient strain OND2017. Transformants isolated on chloramphenicol plates were urease negative.

### Reconstruction of *ureA* promoter to incorporate *tetO* sites and generation of conditional urease mutants

Five *tetO* modified *ureA* promoter constructs *urePtetO*(I-V), containing up to three *tetO* sites, were constructed by SOE PCR (S4 Fig). The primer pairs used to make each *urePtetO* construct are listed in S4 Table. Briefly, a 1 kb fragment upstream, arm I, and a 1.5 kb fragment downstream, arm II, of P_ureA_ were amplified using 26695 genomic DNA as a template. Long primer tails were used to reconstruct the *ureA* promoter region upon fusion of arms I and II by SOE PCR. Primers ureArcat7 and ureArcat8 were used to amplify the final 2.5 kb products, *urePtetO*(I-V), and sequencing confirmed that the modified *ureA* promoters were reconstructed correctly. Natural transformation of the recipient strain OND2017 with *urePtetO* PCR constructs resulted in replacement of the *rpsL-cat* with *urePtetO* and restoration of *ureA*, generating strains X47 *urePtetO*I through X47 *urePtetO*V (OND2018—OND2022). Correct allelic replacement of the resulting Str^r^ transformants was confirmed by colony PCR using primers ureAP1 and ureArcat8 and by sequencing using primer urePseq.

Conditional urease strains were generated by transforming the TetR expressing *H*. *pylori* strain, X47 *mdaB*::*ptetR*4 (OND1987) [32], with the *ureA*::*rpsL-cat* PCR construct to generate the recipient strain OND2026. This urease negative strain was then transformed with each of the five *urePtetO* constructs to generate conditional urease mutant strains X47 *mdaB*::*ptetR*4; *urePtetO*(I-V) (OND1954—OND1958). Transformants were first screened for tetracycline dependent urease expression using the urease phenotype assay and additional characterization was done using the urease activity assay and immunoblot analysis.

### Urease phenotype assay

Urea culture plates (Brucella broth, 7% NCS, 1 mM urea, phenol red 100 mg/l, vancomycin 6 mg/l, pH 6) were used to assay the urease phenotype of *H*. *pylori* clones. The pH of the media was adjusted with 1 M HCl before the addition of NCS and vancomycin. The pH was low enough to observe the colourimetric change of phenol red, from yellow to red, due to the catalytic activity of urease on urea, but not acidic enough to inhibit the growth of urease negative strains. To screen for *tet-*regulated urease activity, transformants and colonies re-isolated from infected animals were replica plated onto CBA plates with or without 50 ng/ml of ATc and cultured for 48 h. Bacteria were then patched onto urea plates and incubated under microaerobic conditions. Urea plates were examined after 16 h of incubation to identify clones that had switched urease phenotype upon exposure to ATc. Localized changes in colour around each growing colony identified urease positive clones. Conditional urease mutant strains grown on CBA plates without ATc remained urease negative, while strains grown on CBA plates with ATc became urease positive (Example S5 Fig).

### Measurement of urease enzymatic activity

The urease activity assay used in this study was adapted from the protocol previously described [28]. Strains were grown on CBA plates without or with 50 ng/ml ATc for two successive passages. Bacteria from 24 h plate cultures were collected and resuspended in cold buffer A (25 mM phosphate buffer, pH 6.8) and standardized to an OD_600_ = 4.0. A 50 μl aliquot of the standardized bacterial suspension was then diluted with 50 μl of buffer B (25 mM phosphate buffer, pH 6.8, 0.2% Tween-20). A 25 μl aliquot of this diluted bacterial suspension was transferred into one well of a 96 well plate, diluted with 150 μl of buffer C (25 mM phosphate buffer, pH 6.8, 250 μM phenol red) and incubated for 5 min at 37°C. A 75 μl aliquot of urea solution (0.5 M) was then added to the well and the absorbance at 560 nm was measured every 72 s for 75 cycles using a POLARstar Omega (BGM Labtech) plate reader. Activity was measured as the rate of change in absorbance over time and expressed as percent of urease activity of the wild-type X47 strain. All urease activity measurements were carried out in triplicate and experiments were repeated at least three times.

### Time course experiments of *tet*-regulation

Bacteria were grown in Heart Infusion (HI) medium supplemented with 10% Newborn Calf Serum (NCS) and vancomycin (6 μg/ml). Cultures were inoculated with bacteria suspended in PBS to give a starting OD_600_ = 0.05, and grown under microaerobic conditions at 37°C and 120 rpm. For induction, *H*. *pylori* cultures were grown to mid-log phase in 10 ml of media. Cultures were induced with 200 ng/ml ATc and bacteria were incubated for another 12 h, with aliquots were taken at indicated time points. For gene silencing, conditional strains were cultured in the presence of 200 ng/ml ATc to mid-log phase. Fresh HI media, with or without 200 ng/ml ATc, was inoculated with pre-induced bacteria (OD_600_ = 0.5) and grown for 12 h, with aliquots taken at indicated time points. Bacterial cells were collected by centrifugation and washed twice with PBS before processing for immunoblot analysis.

### SDS-PAGE and immunoblot analysis

Bacterial whole cell lysates were prepared as previously described [32]. The protein concentration of bacterial cell whole cell lysate samples was determined using the Micro BCA protein assay reagent kit (Pierce) with bovine serum albumin as the standard. Equal amounts of protein for each sample were mixed with 3x SDS-PAGE sample loading buffer, incubated at 95°C for 10 min, and proteins were separated by 10% SDS-PAGE and electrotransferred to a PVDF membrane. For detection of the UreB subunit of urease, mouse anti-UreB (Austral biologicals) was used at a dilution of 1:8000. Secondary antibody rabbit anti-mouse-HRP (Jackson ImmunoResearch Laboratories) was used at a dilution of 1:10,000 and detection of the secondary HRP conjugate was accomplished by chemiluminescence (Sigma) using LAS 3000 (Fujifilm) (software Image reader LAS 3000 V2.2). For loading controls, duplicate gels were run in parallel and stained with Coomaise Brilliant Blue R-250 (S1B Fig, S6 Fig and S7 Fig).

### Animal experiments

Mouse procedures were reviewed and approved by the Institutional Animal Care and the Animal Ethics Committee of the University of Western Australia. 6–7 week old C57BL/6J female mice were challenged once by oral gavage with 200 μl of 1 x 10^9^ CFU/ml of bacteria suspended in HI broth. Groups of infected mice received doxycycline (Dox), anhydrotetracycline (ATc) or no supplement in drinking water containing 5% sucrose. Water was kept in light-protected bottles and changed every three days. Mice were sacrificed at indicated time points and stomachs were removed and homogenized in 1 ml HI using a tissue lyser (Retch). Homogenates were serially diluted and plated out on *H*. *pylori* selective plates (CBA containing 5% Horse blood, Dent, nalidixic acid 10 mg/l and Bacitracin 100 mg/l) to determine the bacterial burden. Were appropriate, re-isolated clones were assayed for *tet*-responsive gene expression.

### Illumina library preparation and sequencing

Preparation of MiSeq library was performed using Illumina Nextera XT DNA sample preparation kit (Illumina, San Diego, CA, USA) as previously described with minor modifications [64]. In brief, 1 ng of genomic DNA was fragmented in 5 μl of Amplicon Tagment Mix and 10 μl of Tagment DNA buffer. Tagmentation reaction was performed by incubation at 55°C for 10 min followed by neutralisation with 5 μl of Neutralise Tagment Buffer for 5 min. Tagmented DNA (25 μl) was indexed in a 50 μl limited-cycle PCR (12 cycles) as outlined in the Nextera XT protocol and subsequently purified using 25 μl of AMPure XP beads (Beckman Coulter Inc, Australia). The fragment size distribution of the purified DNA was analysed utilising a LabChip GXII 2100 Bioanalyser. DNA libraries were adjusted to 2 nM, pooled in equal volumes and then denatured with 0.2 N NaOH according to the Nextera protocol. The libraries were sequenced using 2 × 300 paired-end protocols on an Illumina MiSeq instrument (MiSeq Reagent Kit v3 for 600 cycles). The draft genome sequence of *H*. *pylori* OND1954 has been deposited at DDBJ/ENA/GenBank under the accession MVFB00000000. The version described in this paper is version MVFB01000000. All raw sequence data generated in this study have been submitted to Sequence Read Archives (SRA) database with accession numbers listed in S5 Table.

### Sequence data de novo assembly, annotation, reference mapping and variant calling

The generated MiSeq reads of *H*. *pylori* strain OND1954 was assembled using SPAdes genome assembler (version 3.8.2) with careful option [65]. The draft genome sequence was subsequently annotated using Prokka (version 1.11) with Swiss-Prot, Pfam (release 30.0), TIGRFAMs (release 15.0) and Superfamily (version 1.75) databases [66–70]. The annotation features are available in S1 File. The raw reads of OND1954-derivative strains were trimmed and mapped against the annotated draft genome using Bowtie2 on Geneious R7 platform [71, 72]. Variants were called using the following parameters: minimum coverage = 10 and minimum variant frequency = 0.7.

### Statistical analysis

For mouse colonization assays (where n ≥ 5) the Mann-Whitney unpaired two-tailed test was used to compare colonization loads and the two sided Fisher’s exact test was used to compare infection rates. Bonferroni correction was used for multiple pairwise testing. Statistical analysis was performed using GraphPad Prism version 7 for Windows, (GraphPad Software) and Stata Statistical Software (StataCorp. 2015. *Release 14*. College Station, TX: StataCorp LP.).

## Supporting information

S1 Fig TetR silencing of *urePtetO* in *H*. *pylori*. UreB protein was detected in *H*. *pylori* strains harbouring *urePtetO* and expressing TetR under the control P_flaA_ (OND1954—OND1958). Bacteria were cultured on standard CBA plates or CBA plates containing 50 ng/ml ATc for 48 h and fresh bacteria cultures were used to prepare whole cell lysates. Equal amount of protein was loaded into each lane and separated on a 10% SDS–PAGE gel. (A)The *urePtetO* construct is specified under the bars. UreB protein could not be detected in samples from bacteria grown in the absence of ATc. UreB expression was strongly induced in strains harbouring *urePtetO*I, *urePtetO*II and *urePtetO*V. Induction of UreB expression was weaker in strains harbouring *urePtetO*III and *urePtetO*IV. (B) Coomassie stain of duplicate gel.
(TIF)

S2 Fig *H*. *pylori* X47 tolerance to Dox *in vivo*. (A). Mice were orally challenged with wild-type X47 strain and supplemented with a range of Dox concentrations (1, 10, 100 and 1000 mg/l) in their drinking water. Bars represent median bacterial load per group and points plotted represent colonization density for each individual animal. Detection limit was < 50 CFU per stomach (dotted horizontal line). Gastric specimens without *H*. *pylori* re-isolation are shown as null. (B) Urease expression in conditional urease mutant strain OND1954 was induced with 50 ng/ml ATc for 48 h prior to oral challenge. Mice were orally challenged with wild-type X47 strain or pre-induced OND1954 and supplemented with a range of Dox concentrations (1–20 mg/l) in their drinking water. Animals were sacrificed one week after oral challenge. Bars represent median bacterial load per group (n = 3) and points plotted represent colonization density for each individual animal. Detection limit was < 50 CFU per stomach (dotted horizontal line). Gastric specimens without *H*. *pylori* re-isolation are shown as null.
(TIF)

S3 Fig Diagram of PCR construction strategy for *ure*::*rpsL-cat*. (TIF)

S4 Fig Diagram of PCR construction strategy for *urePtetO*. (TIF)

S5 Fig Assay for urease activity using acidified urea plates. To screen for *tet*-regulated urease activity, *H*. *pylori* clones were replica plated onto CBA plates without (control) or with 50 ng/ml of ATc and cultured for 48 h. Bacteria were then patched onto urea plates and incubated under microaerobic conditions. An example from testing clones of strain OND1954 is provided. Change in colour due to urease activity is shown after 0.25 h, 2 h and 16 h of incubation. Conditional urease mutant strains grown on CBA plates without ATc remained urease negative, while strains grown on CBA plates with ATc became positive for urease activity.
(TIF)

S6 Fig Loading control for Fig 1C. Coomassie stain of duplicate gel for UreB expression in X47 *urePtetO*(I-V) strains compared to wild-type X47.
(TIF)

S7 Fig Loading controls for Fig 2B and 2C time course experiments. Coomassie stain of duplicate gels for time course of TetR-controlled expression of UreB in conditional urease mutants OND1954 and OND1958.
(TIF)

S1 Table CDS mutations identified in *tet*-escape mutants. (XLSX)

S2 Table Strains used in this study. (DOCX)

S3 Table Oligonucleotide primers used in this study. (DOCX)

S4 Table Oligonucleotide primer pairs used to generate *urePtetO* constructs. (DOCX)

S5 Table Strain SRA accession no. (DOCX)

S1 File OND1954 genome sequence with annotated features. (GFF)
