# Supplementary figures and images for: Helicobacter pylori gene silencing in vivo demonstrates urease is essential for chronic infection
Source: PLoS Pathog. 2017 Jun 23;13(6):e1006464. doi: 10.1371/journal.ppat.1006464 (PMC5500380; doi:10.1371/journal.ppat.1006464)

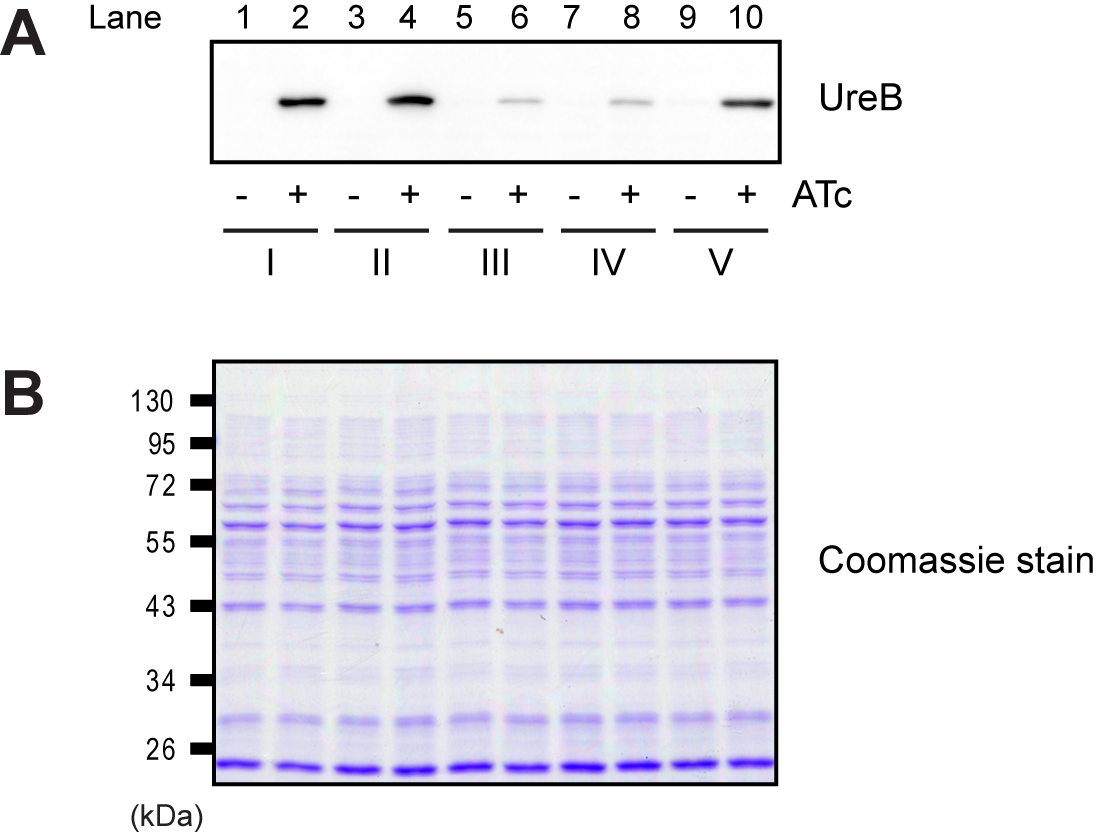

Supplement: S1 Fig — UreB protein was detected in H. pylori strains harbouring urePtetO and expressing TetR under the control PflaA (OND1954—OND1958). Bacteria were cultured on standard CBA plates or CBA plates containing 50 ng/ml ATc for 48 h and fresh bacteria cultures were used to prepare whole cell lysates. Equal amount of protein was loaded into each lane and separated on a 10% SDS–PAGE gel. (A)The urePtetO construct is specified under the bars. UreB protein could not be detected in samples from bacteria grown in the absence of ATc. UreB expression was strongly induced in strains harbouring urePtetOI, urePtetOII and urePtetOV. Induction of UreB expression was weaker in strains harbouring urePtetOIII and urePtetOIV. (B) Coomassie stain of duplicate gel. (TIF) [file ppat.1006464.s001.tif]

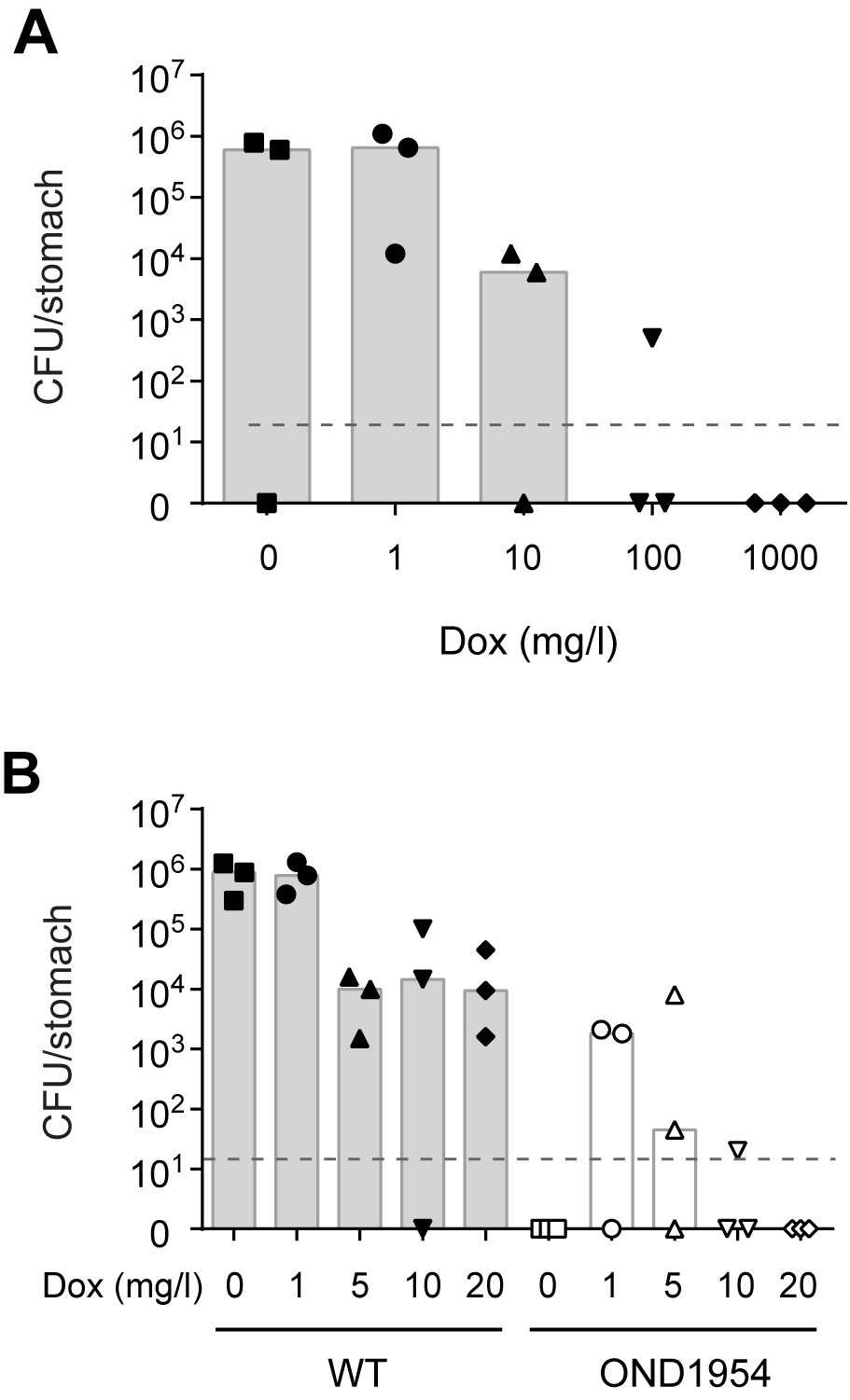

Supplement: S2 Fig — (A). Mice were orally challenged with wild-type X47 strain and supplemented with a range of Dox concentrations (1, 10, 100 and 1000 mg/l) in their drinking water. Bars represent median bacterial load per group and points plotted represent colonization density for each individual animal. Detection limit was < 50 CFU per stomach (dotted horizontal line). Gastric specimens without H. pylori re-isolation are shown as null. (B) Urease expression in conditional urease mutant strain OND1954 was induced with 50 ng/ml ATc for 48 h prior to oral challenge. Mice were orally challenged with wild-type X47 strain or pre-induced OND1954 and supplemented with a range of Dox concentrations (1–20 mg/l) in their drinking water. Animals were sacrificed one week after oral challenge. Bars represent median bacterial load per group (n = 3) and points plotted represent colonization density for each individual animal. Detection limit was < 50 CFU per stomach (dotted horizontal line). Gastric specimens without H. pylori re-isolation are shown as null. (TIF) [file ppat.1006464.s002.tif]

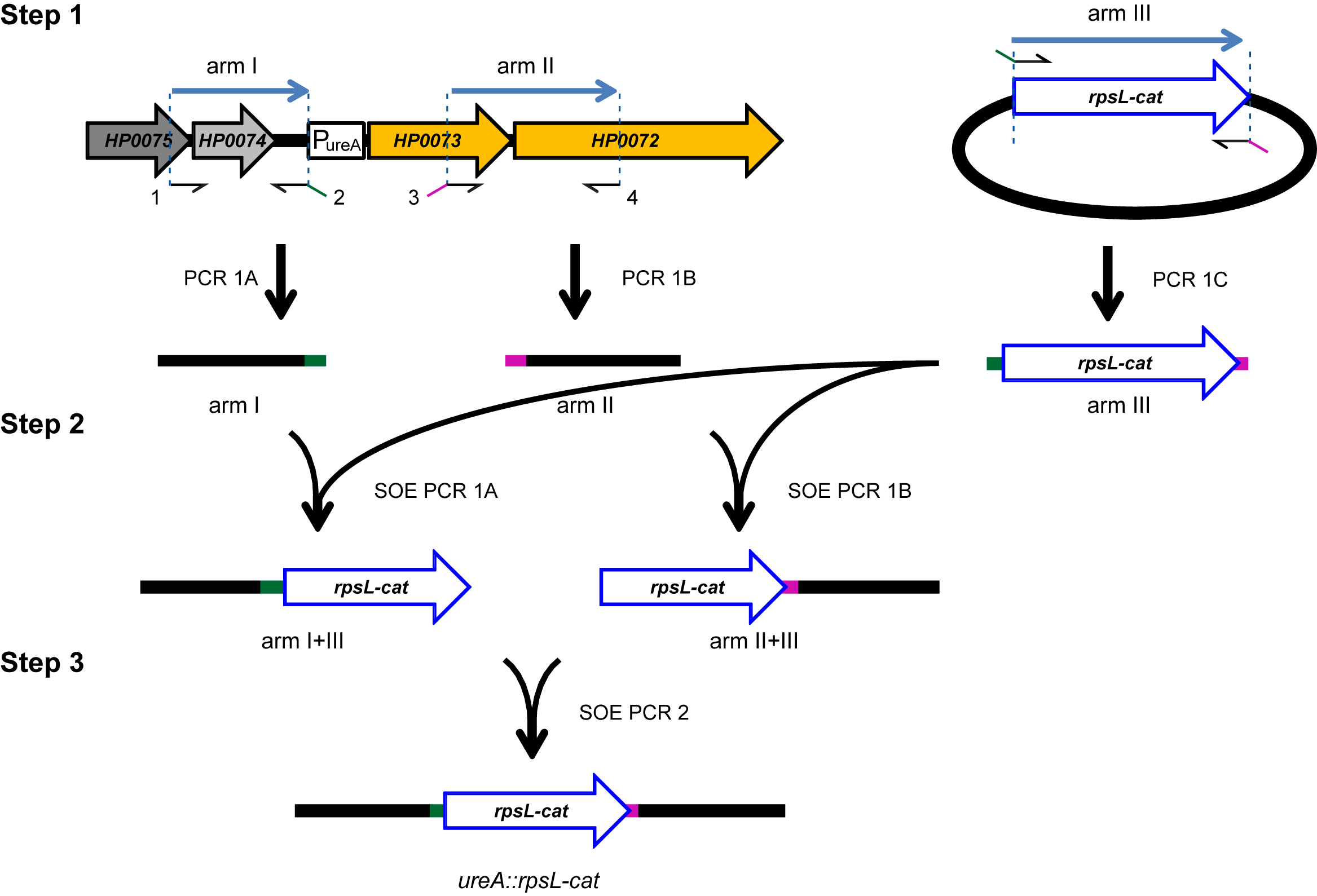

Supplement: S3 Fig — (TIF) [file ppat.1006464.s003.tif]

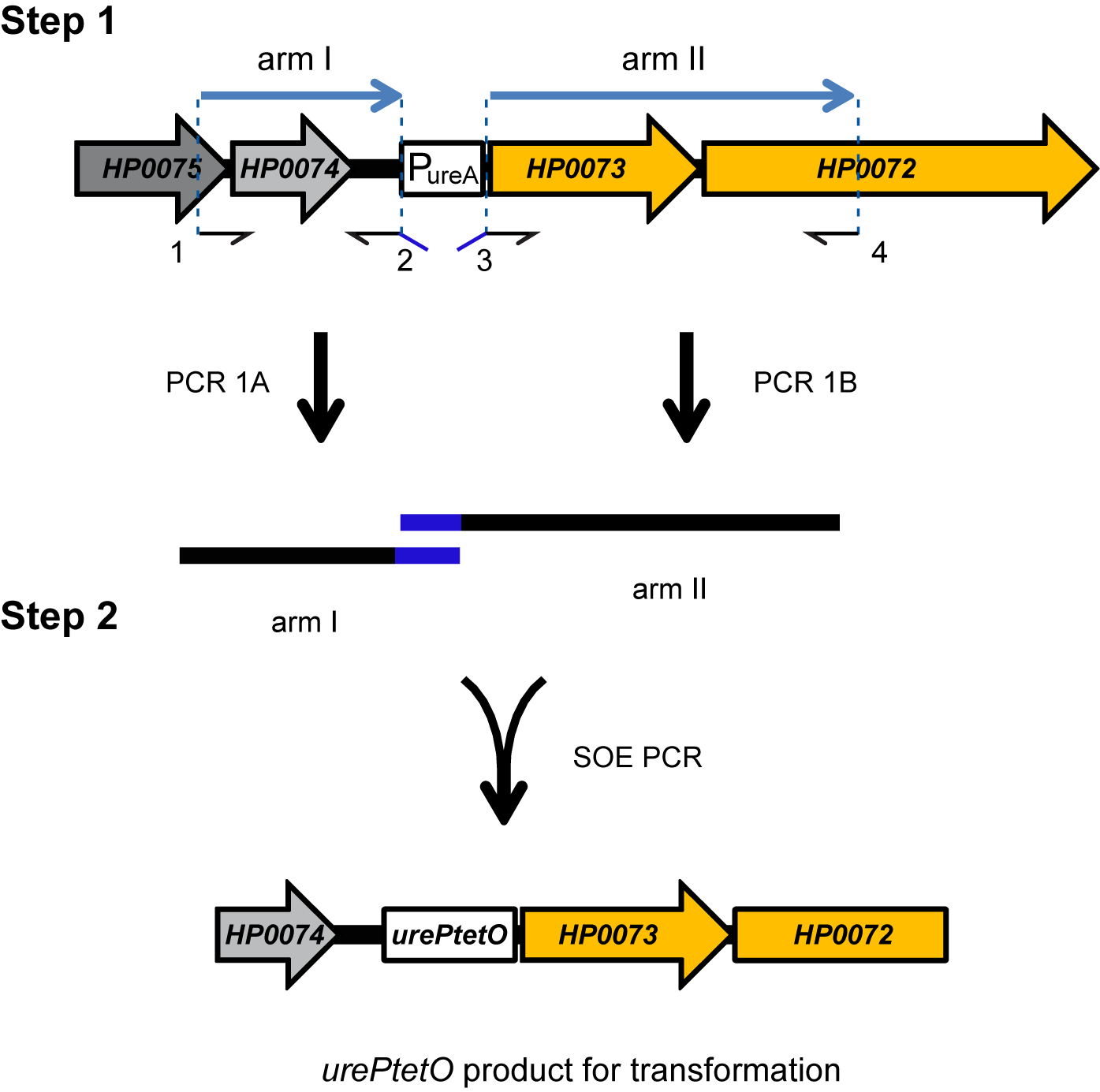

Supplement: S4 Fig — (TIF) [file ppat.1006464.s004.tif]

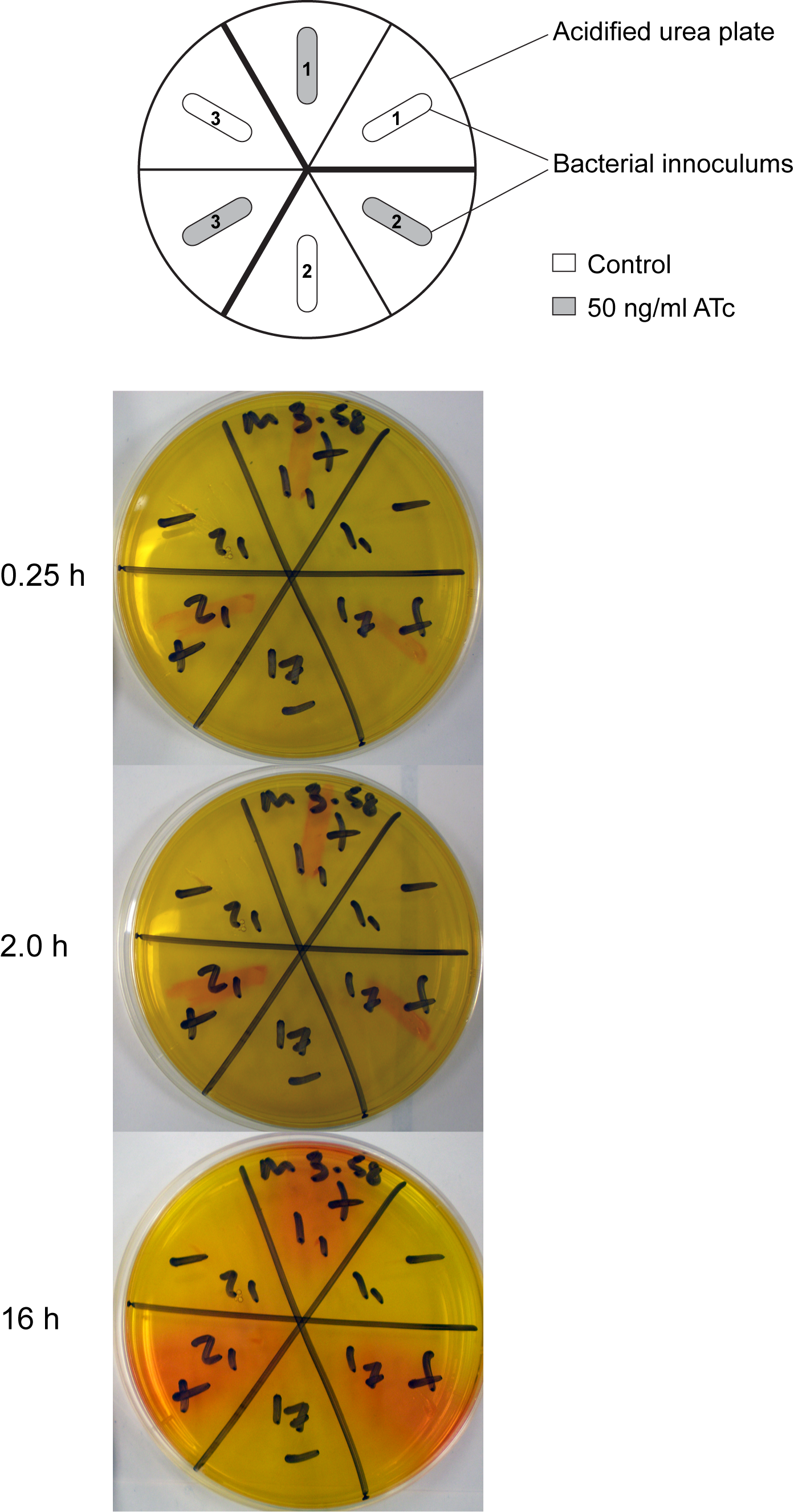

Supplement: S5 Fig — To screen for tet-regulated urease activity, H. pylori clones were replica plated onto CBA plates without (control) or with 50 ng/ml of ATc and cultured for 48 h. Bacteria were then patched onto urea plates and incubated under microaerobic conditions. An example from testing clones of strain OND1954 is provided. Change in colour due to urease activity is shown after 0.25 h, 2 h and 16 h of incubation. Conditional urease mutant strains grown on CBA plates without ATc remained urease negative, while strains grown on CBA plates with ATc became positive for urease activity. (TIF) [file ppat.1006464.s005.tif]

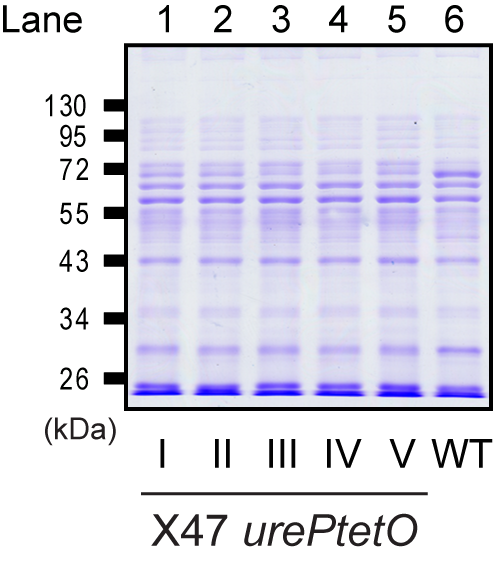

Supplement: S6 Fig — Coomassie stain of duplicate gel for UreB expression in X47 urePtetO(I-V) strains compared to wild-type X47. (TIF) [file ppat.1006464.s006.tif]

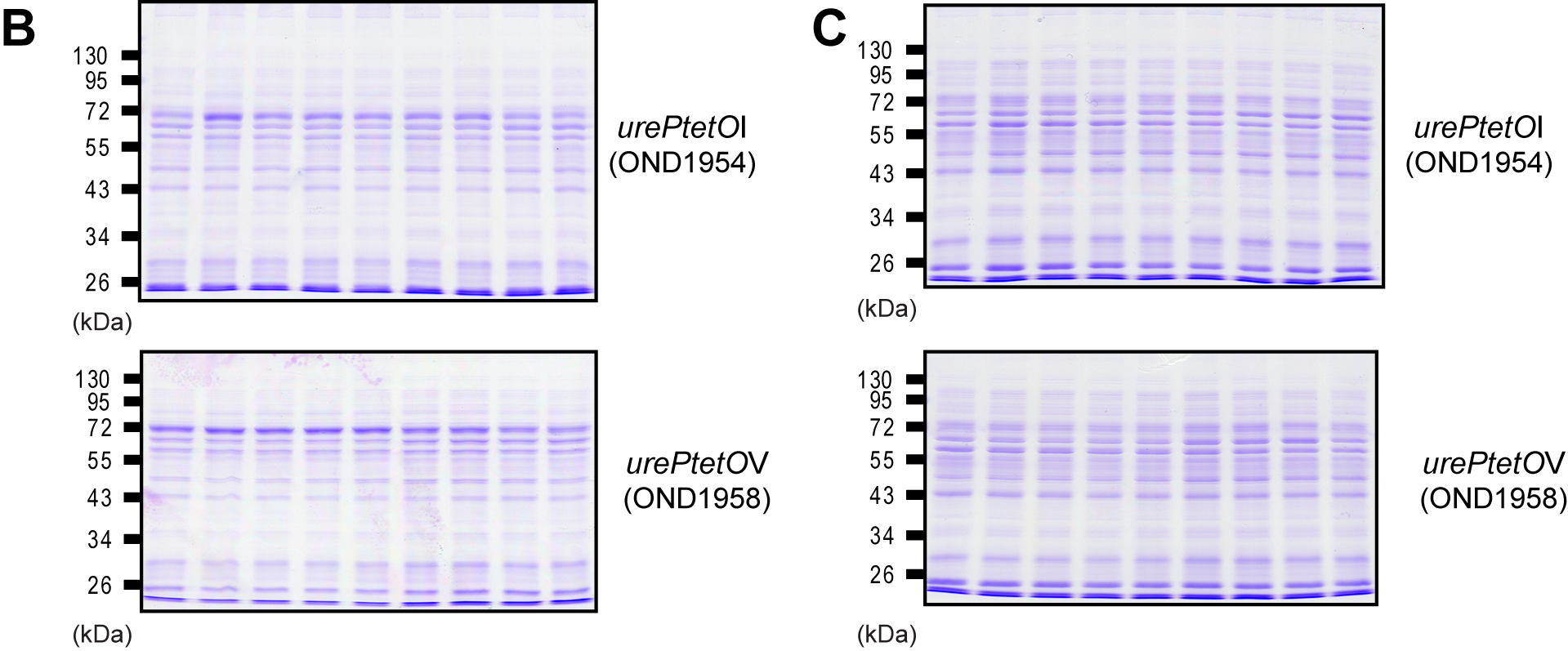

Supplement: S7 Fig — Coomassie stain of duplicate gels for time course of TetR-controlled expression of UreB in conditional urease mutants OND1954 and OND1958. (TIF) [file ppat.1006464.s007.tif]
