# Supplementary material for: Helicobacter pylori gene silencing in vivo demonstrates urease is essential for chronic infection
Source: PLoS Pathog. 2017 Jun 23;13(6):e1006464. doi: 10.1371/journal.ppat.1006464 (PMC5500380; doi:10.1371/journal.ppat.1006464)
Supplement: S2 Table — (DOCX) [file ppat.1006464.s009.docx]

**S2 Table** Strains used in this study

| ***H. pylori* strain name** | | **Strain ID** | **Description** | | | **Source or reference** |  |
| --- | --- | --- | --- | --- | --- | --- | --- |
| X47 | | OND79 | wild-type strain, also known as X47-2AL, naturally resistant to streptomycin | | | [1] |  |
| X47 Δ*pureA::rpsL-cat* | | OND2017 | *ureA* and upstream promoter replaced with *rpsL-cat* | | This work | |  |
| X47 *urePtetO*I | | OND2018 | Two *tetO* operators located in P_ureA_, flanking -35 region | | This work | |  |
| X47 *urePtetO*II | | OND2019 | One *tetO* operator located in in P_ureA_, between -35 and -10 regions | | This work | |  |
| X47 *urePtetO*III | | OND2020 | Three *tetO* operators located in in P_ureA_, flanking -35 and -10 regions | | This work | |  |
| X47 *urePtetO*IV | | OND2021 | Two *tetO* operators located in P_ureA_, flanking -10 region | | This work | |  |
| X47 *urePtetO*V | | OND2022 | One *tetO* operators located in P_ureA_, downstream of -10 region | | This work | |  |
| X47 *mdaB::ptetR*4 | | OND1987 | P*_flaA_-tetR* inserted between HP630 and HP631 | | [2] | |  |
| X47 *mdaB::ptetR*4; Δ*pureA::rpsL-cat* | | OND2026 | P_flaA_*-tetR* inserted between HP630 and HP631; *ureA* and upstream promoter replaced with *rpsL-cat* | | This work | |  |
| X47 *mdaB::ptetR*4*; urePtetO*I | | OND1954 | P_flaA_*-tetR* inserted between HP630 and HP631; two *tetO* operators flanking -35 region of P_ureA_ | | This work | |  |
| X47 *mdaB::ptetR*4*; urePtetO*II | | OND1955 | P_flaA_*-tetR* inserted between HP630 and HP631; one *tetO* operator located between -35 and -10 of P_ureA_ | | This work | |  |
| X47 *mdaB::ptetR*4*; urePtetO*III | | OND1956 | P_flaA_*-tetR* inserted between HP630 and HP631; three *tetO* operators flanking -35 and -10 regions of P_ureA_ | | This work | |  |
| X47 *mdaB::ptetR*4*; urePtetO*IV | | OND1957 | P_flaA_*-tetR* inserted between HP630 and HP631; two *tetO* operators flanking -10 region of P_ureA_ | | This work | |  |
| X47 *mdaB::ptetR*4*; urePtetO*V | | OND1958 | P_flaA_*-tetR* inserted between HP630 and HP631; one *tetO* operator downstream of -10 region of P_ureA_ | | This work | |  |
| X47 *mdaB::ptetR*4; *urePtetO*I-MP, pooled isolates A-E | | OND3241 (A-E) | P_flaA_*-tetR* inserted between HP630 and HP631; *ureA* promoter replaced with *urePtetO*I; strains re-isolated from C57BL/6J mice | | This work | |  |
|  |  | |  |  | | | |

**Supporting References**

1. Ermak TH, Giannasca PJ, Nichols R, Myers GA, Nedrud J, Weltzin R, et al. Immunization of mice with urease vaccine affords protection against *Helicobacter pylori* infection in the absence of antibodies and is mediated by MHC class II-restricted responses. J. Exp. Med. 1998;188(12):2277-88.

2. Debowski AW, Verbrugghe P, Sehnal M, Marshall BJ, Benghezal M. Development of a tetracycline-inducible gene expression system for the study of *Helicobacter pylori* pathogenesis. Appl. Environ. Microbiol. 2013;79(23):7351-9.
