# Supplementary material for: Helicobacter pylori gene silencing in vivo demonstrates urease is essential for chronic infection
Source: PLoS Pathog. 2017 Jun 23;13(6):e1006464. doi: 10.1371/journal.ppat.1006464 (PMC5500380; doi:10.1371/journal.ppat.1006464)
Supplement: S3 Table — (DOCX) [file ppat.1006464.s010.docx]

**S3 Table** Oligonucleotide primers used in this study

| **Name** | **Sequence (5′–> 3′)** | **Function of PCR product** |
| --- | --- | --- |
| ureArcat1 | CGTTAGTGTTAGAAAGCAAGCAG | Inactivation of *ureA* with *rpsL-cat* |
| ureArcat2 | CATAGTTATAAAGCATCTTTAAAATGAATTAGTGTTATATCTTTGAAG |  |
| ureArcat3 | CTGAATAAATAAAATCCTAAAAATGTTGGCGACAGACCGGTTC |  |
| ureArcat4 | ACGCATGATTGATTGCAGAAGGAG |  |
| ureArcat5 | CACTAATTCATTTTAAAGATGCTTTATAACTATGGATTAAACAC |  |
| ureArcat6 | CCAACATTTTTAGGATTTTATTTATTCAGCAAGTCTTG |  |
| ureArcat7 | CCAAAGCCTAGTGAATTTGAATGTC |  |
| ureArcat8 | ATCGCACCAGCTTCAATTTGATC |  |
| ureAtetO1 | GATGTAATTGTAGCATCTCTATCACTGATAGGGATTAACATCTCTATCACTGATAGGGATATTATTTAAAATGAATTAGTGTTATATCTTTGAAG | Reconstruction of *ureA* promoter with *tetO* sites |
| ureAtetO2 | CAGTGATAGAGATGCTACAATTACATCCAACCTTG |  |
| ureAtetO3 | GATGTAATTGTAGCATCTCTATCACTGATAGGGATTAACAGAAGCGTTCATTAAC |  |
| ureAtetO4 | GATAGGGATGTAATTGTAGCATCTCTATCACTGATAGGGATTAACATCTCTATCACTGATAGGGATATTATTTAAAATGAATTAGTGTTATATCTTTGAAG |  |
| ureAtetO5 | GAGATGCTACAATTACATCCCTATCAGTGATAGAGATGTCTTCAAGGAAAAACACTTTAAGAATAGG |  |
| ureAtetO6 | GACATCTCTATCACTGATAGGGATGTAATTGTAGCATCTCTATCACTGATAGGGATTAACAGAAGCGTTCATTAAC |  |
| ureAtetO7 | CATCCCTATCAGTGATAGAGATGTCTTCAAGGAAAAACACTTTAAGAATAGG |  |
| ureAtetO8 | GACATCTCTATCACTGATAGGGATGTAATTGTAGCAATGTTTTGATTTACTAAG |  |
| urePseqF | CGTTTTCCTTGCTCAGTTTTTTAGAG | Sequencing *ureA* promoter region |
| urePseqR | GTCTTTTTACCAGCTCTCGCTTC |  |
| trpAseqF | TTCCACCGCCTTCAAAGTCAAG | Sequencing *tetR* in *trpA* locus |
| trpAseqR | CAGCTTGCCATTTTGGCTATT |  |
