# Supplementary material for: Helicobacter pylori gene silencing in vivo demonstrates urease is essential for chronic infection
Source: PLoS Pathog. 2017 Jun 23;13(6):e1006464. doi: 10.1371/journal.ppat.1006464 (PMC5500380; doi:10.1371/journal.ppat.1006464)
Supplement: S4 Table — (DOCX) [file ppat.1006464.s011.docx]

**S4 Table** Oligonucleotide primer pairs used to generate *urePtetO* constructs

| **Construct** | **arm I - upstream** | **arm II - downstream** |
| --- | --- | --- |
| *urePtetO*I | ureArcat1 & ureAtetO1 | ureAtetO2 & ureArcat4 |
| *urePtetO*II | ureArcat1 & ureAtetO3 | ureAtetO2 & ureArcat4 |
| *urePtetO*III | ureArcat1 & ureAtetO4 | ureAtetO5 & ureArcat4 |
| *urePtetO*IV | ureArcat1 & ureAtetO6 | ureAtetO7 & ureArcat4 |
| *urePtetO*V | ureArcat1 & ureAtetO8 | ureAtetO7 & ureArcat4 |
