# Supplementary material for: Helicobacter pylori gene silencing in vivo demonstrates urease is essential for chronic infection
Source: PLoS Pathog. 2017 Jun 23;13(6):e1006464. doi: 10.1371/journal.ppat.1006464 (PMC5500380; doi:10.1371/journal.ppat.1006464)
Supplement: S5 Table — (DOCX) [file ppat.1006464.s012.docx]

**S5 Table** Strain SRA accession no

| **Strain** | **SRA accession no.** |
| --- | --- |
| OND1954 | SRX2557411 |
| 15A1 | SRX2557410 |
| 15A2 | SRX2557409 |
| 15A3 | SRX2557408 |
| 15A4 | SRX2557407 |
| 15B1 | SRX2557406 |
| 15B2 | SRX2557405 |
| 15B3 | SRX2557404 |
| 15C1 | SRX2557403 |
| 15C2 | SRX2557402 |
| 15C3 | SRX2557401 |
| 15D1 | SRX2557400 |
| 15D2 | SRX2557399 |
| 15D3 | SRX2557398 |
| 15D4 | SRX2557397 |
| 15E1 | SRX2557396 |
| 15E2 | SRX2557395 |
| 15E3 | SRX2557394 |
| 15E4 | SRX2557393 |
| 17A1 | SRX2557392 |
| 17A2 | SRX2557391 |
| 17A3 | SRX2557390 |
| 17A4 | SRX2557389 |
| 17B1 | SRX2557388 |
| 17B2 | SRX2557387 |
| 17B3 | SRX2557386 |
| 17D1 | SRX2557385 |
| 17D2 | SRX2557384 |
| 17D3 | SRX2557383 |
| 17D4 | SRX2557382 |
| 16A | SRX2557381 |
| 16B | SRX2557380 |
| 16L | SRX2557379 |
| 18A | SRX2557378 |
| 18B | SRX2557377 |
| OND3241A | SRX2557376 |
| OND3241B | SRX2557375 |
| OND3241C | SRX2557374 |
| OND3241D | SRX2557373 |
| OND3241E | SRX2557371 |
